# Validity and Reliability Study of the Workplace Violence Scale in Healthcare (TYPE 2 Violence) in Turkish

**DOI:** 10.3390/healthcare13070729

**Published:** 2025-03-25

**Authors:** Elif Yöyen, Tülay Güneri Barış

**Affiliations:** 1 Department of Psychology, Faculty of Humanities and Social Sciences, Sakarya University, Sakarya 54050, Türkiye; 2 Department of Health Sciences, Institute of Business Administration, Sakarya University, Sakarya 54050, Türkiye

**Keywords:** workplace violence, violence in health, scale development, validation studies, reliability

## Abstract

**Background:** Workplace violence refers to acts that occur inside or outside the workplace, ranging from verbal harassment, bullying, threats and physical assaults against workers to homicide. Workplace violence in health care settings is an incident involving verbal, physical or sexual assault against healthcare workers by patients, their relatives or others that poses a threat to healthcare workers. Violence in healthcare settings is a significant public health problem, not only for the victims of violence, but also for society because of its direct and indirect, short and long-term effects. **Objectives:** The aim of this study is to adapt the Workplace Violence Scale in Healthcare to Turkish, to conduct validity and reliability studies, and to determine its psychometric properties in order to overcome the problems faced by health policy makers in assessing workplace violence and to ensure that they implement appropriate interventions. **Methods:** In the research in which 191 healthcare workers were included in the pilot study and 627 healthcare workers in the main sample, data were collected using the Sociodemographic Data Form and the Workplace Violence Scale in Healthcare. SPSS 25.0 and AMOS 25.0 programs were used to analyse the data. In the scale validity and reliability study stage, Explanatory Factor Analysis and Confirmatory Factor Analysis methods were used after the language and content validity analyses. **Results:** The Cronbach alpha coefficient of the scale was found to be 0.946, and it was observed that the CR values of the scale consisting of five subdimensions and 37 items were over 0.70 and the AVE values were over 0.50. At the same time, in order to reveal the stability of the scale over time, the test-retest method was applied, and it was seen that the correlation coefficients obtained were 0.97 for the whole scale and between 0.80 and 0.94 for its subdimensions, indicating an excellent level of reliability. **Conclusions:** As a result of this study, it was accepted that the Workplace Violence Scale in Healthcare, developed with five subdimensions (frequency of workplace violence, impact of workplace violence on the individual, reasons for not reporting workplace violence legally, risk factors increasing workplace violence and workplace violence prevention strategies) and 37 items, can be used as a comprehensive and standard measurement tool that evaluates to measure workplace violence in healthcare settings. With this scale, future studies will be able to determine the type of violence (physical/verbal) that patients and their relatives use against healthcare professionals, how often healthcare professionals are exposed to violence, and how they, their families and social circles are affected physically and psychologically by the violence they experience (psychologically and socially). In addition, the results from the sections of the scale that ask about the risk factors for violence, the reasons why violence is not legally reported, and the practices used to prevent violence can guide health and legal policy makers.

## 1. Introduction

Violence has a complex structure due to the fact that it contains many social, psychological, biological, economic, cultural, political and religious elements, and it is extremely difficult to express it with a single definition. When examining the studies that have been conducted, it is found that the terms violence and aggression are generally used interchangeably and are definitions that cause confusion [1]. However, aggression contains elements of violence, has offensive, disruptive and obstructive purposes to the person it is directed at and expresses the more mental and psychological state of the person, while violence refers to the behaviour of aggression turned into action, with movement shown [2].

The variety of behaviours that can be included under the heading of workplace violence, as well as the confusion between the concepts of aggression and violence, makes it difficult to define workplace violence. It is quite difficult to draw the boundaries of acceptable behaviour and to both recognise and define this phenomenon [3]. Workplace violence refers to acts that take place inside or outside the workplace, ranging from verbal harassment, bullying, threats and physical assaults to homicides against workers. According to the WHO report published in 2002, workplace violence is defined as overt or covert defiance, threats and attacks against the safety and health of workers, including when they travel to and from work [4]. Workplace violence includes all types of behaviours and events that are intentional and designed to cause physiological and/or psychological harm to employees in relation to their work. Although studies conducted until recently focused more on physical violence because its definition was clearer and more concrete, the profile of violence has now changed, and equal importance has begun to be given to non-physical violent behaviours. A multicentre study conducted in 2002 reported that the prevalence of non-physical violence is much higher than that of physical violence and that the psychological problems experienced by individuals as a result of exposure to violence lead to many psychosomatic illnesses [5]. The International Labour Organization (ILO) defines workplace violence as any behaviour in which an individual is subjected to violence, harm or threats during or in relation to their work that is far from reasonable behaviour. Therefore, this definition includes different forms of behaviour such as physical aggression, verbal abuse, intimidation, sexual harassment, and racial or psychological harassment [6,7].

Although it is known that violence is embedded in the cultural, social, economic and political conditions of society and in people’s stereotypes and attitudes, workplace violence is one of the problems that remain valid for all countries. Although there is no single and clear definition of workplace violence due to its subjective nature and diversity of perceptions [8], the European Commission has defined this concept as threats or attacks against individuals at their workplace or during their commute to work [9,10], the World Health Organization as verbal, physical or sexual assaults against the physical or psychological health of health workers by patients and/or their relatives or other individuals that pose a threat to the worker [11], and the situation consisting of verbal, physical and sexual assaults against health workers by individuals such as colleagues, patients and their relatives or third parties that pose a threat to the parties [12].

Regardless of the uncertainty surrounding the precise definition of workplace violence, it is clear that it is a constant threat to healthcare workers. Compared to other professions, healthcare workers are at higher risk of various types of workplace violence [13]. Studies have shown that workplace violence is particularly prevalent in the public and service sectors but is a problem in almost all sectors. According to a survey conducted by the International Committee of the Red Cross (ICRC) in 2020, there were more than 600 incidents of workplace violence against healthcare workers in 40 countries between February and July, the highest rate of violence in healthcare in the last 20 years [14]. Furthermore, some studies have reported that the incidence of violence in public healthcare is higher than in the private healthcare sector [15], while others have reported no difference in the extent of violence experienced between public and private healthcare institutions [16]. Despite the uncertainty about the prevalence of workplace violence in public and private healthcare settings, what is clear is that healthcare workers are more likely to be assaulted at work than prison guards, police officers, transport workers, and retail or banking workers [17], and that healthcare is in fact the sector with the highest rates of workplace violence [9]. The majority of injuries resulting from workplace assaults that require days away from work occur in healthcare settings [18].

The difficulty of classifying workplace violence, as well as the difficulty of defining it, is also evident in the literature. In the classification developed by the University of Iowa Injury Prevention Research Center (UIIPRC) [19] and the California Occupational Safety and Health Administration [8], workplace violence against healthcare workers is classified as Type II violence. Type II violence is violence perpetrated by individuals who have a relationship with the institution or organisation but are not employed by the same employer. It is the most common form of workplace violence, and the aggressor has a direct or indirect relationship with the institution. The perpetrator is a third party such as a client, patient, student or prisoner, depending on the sector studied [20]. It usually occurs during normal working hours in the workplace. All occupational groups that have frequent contact with people, especially healthcare workers, teachers and social workers, are at higher risk of this type of violence [21]. According to this classification, healthcare workers are mostly exposed to Type II violence, but they are also affected by other types of violence in different forms [22].

The etiological factors of workplace violence in health institutions include personal, organisational and social reasons [23], the cultural structure of society, the insufficient functioning of the judicial mechanism, incomplete or undesirable decisions of the laws, complicated structures of health service areas, the rapid increase in the population in need of health services, insufficient investment in health, insufficient central organisation in health institutions, insufficient sanctions [24], high workload, overcrowded environment due to insufficient number of health workers, hasty visits, mechanised processes, insufficient medical knowledge and mental distress of patients [25], insufficient training of staff in dealing with violence, inadequate communication and an insufficient number of security guards [26]. In addition, night work by healthcare workers has been shown to be a risk factor. A recent study conducted in Italy on workplace violence in emergency services revealed a relationship between night work and the occurrence of workplace violence and suggested that the impact of night shifts worked by health workers should be considered as a strategic way to approach the issue of workplace violence [27].

Many studies have been carried out to analyse the risk factors for violence. According to the study conducted by Tanalı and colleagues, the reason for violence in healthcare is the inadequacy of sanctions against the perpetrators of violence and the fact that doctors are blamed for all problems. The same study identified the lack of health literacy in society as another risk factor. Dağ and Baysal examined the role of the media in healthcare violence in their 2017 study and reported that the way news about violence is reflected in the media plays an important role in influencing society’s perception of violence; the expressions of news language are more prominent in people’s minds [28]. Another study found that the main reason for violent incidents in emergency services is that patients are not sufficiently informed about their conditions [29]. Other factors generally cited in the literature are gender, environmental conditions, substance use, feelings of disappointment, refusal of service, overcrowding and lack of staff training [6]. A 2018 study questioned the causes of violence from a societal perspective and found that 20% of participants believed that employees deserved violence and that the reason for this was that they did not care enough about patients [24]. Similarly, in another study in 2022, 39.0% of participants reported that they thought healthcare professionals sometimes deserved violence [30]. The Oregon Association of Hospitals and Health Systems (OAHHS) classified risk factors for workplace violence into four categories: clinically related risk factors, social and economic risk factors, environmental risk factors, and organisational factors [31]. In a report prepared by the Parliamentary Research Commission in 2013, the causes of violence against healthcare professionals in Turkey were categorised under four main headings. These are the characteristics of the parties (service providers and service recipients), their interactions and communication factors, organisational/institutional factors, and environmental and social factors [32].

Regardless of its definition, classification and risk factors, workplace violence is a global public health problem [33]. A meta-analysis study conducted in 2024 with the participation of 139,533 intensive care unit workers from 32 countries reported that the average frequency of violence was 31% for physical violence, 57% for verbal violence, and 12% for sexual violence [34]. A systematic analysis study conducted by Liu et al. reported that the incidence of workplace violence against healthcare workers worldwide was 61.9%, with non-physical violence in first place at 42.5%, physical violence in second place at 24.4%, and sexual harassment in third place at 12.4% [35]. In 2019, the International Health Safety and Security Foundation (IAHSSF) reported that the rate of assaults on healthcare workers had increased from 9.3% to 11.7% [36], and that there had been an increase in workplace violence rates in the US between 2012 and 2015 [37]. An International Labour Organization (ILO) study of six countries found that 67.2% of healthcare workers in Australia, 75.8% in Bulgaria, 54% in Thailand, 46.7% in Brazil, 61% in South Africa and 60% in Portugal had been subjected to physical or psychological violence at least once in the previous year [32]. Studies have shown that healthcare workers are sixteen times more likely to experience violence in the workplace than people working in other sectors [38], and that the areas where violent incidents occur most frequently are outpatient clinics (51.9%) and emergency departments (24.4%) [28]. Verbal violence occurs most frequently in the triage areas of emergency departments [39], and a systematic review of 331,544 participants reported that the 12-month prevalence of any form of workplace violence among healthcare workers worldwide was 61.9% [40].

Violence in healthcare settings has short- and long-term effects at the individual, organisational and societal levels. It also has many overt and covert effects on the safety, well-being and health of workers [41]. While the psychological and physical damage suffered by the assaulted healthcare worker is more short-term and tangible in terms of consequences, the reduction in quality of patient care and financial problems are difficult to identify and compensate for. In this context, it can be said that workplace violence in healthcare settings has serious psycho-socioeconomic consequences for healthcare workers, service delivery, healthcare organisations and society: The consequences of workplace violence, from the moment of the act and afterwards, affect not only the individual healthcare worker but also their family members and those involved in the worker’s social relationships. The emotions experienced by workers exposed to physical and/or psychological violence include physical and psychological problems such as anger, fear, long-term burnout, depression, anxiety, withdrawal from social relationships, nutritional and sleep disturbances [42], and organisational consequences such as repeated absenteeism or tardiness as a result of demotivation, increased staff turnover, reduced productivity, increased insurance costs and reduced institutional commitment [43].

Although it is known that violence is embedded in the cultural, social, economic and political conditions of society and in people’s stereotypes and attitudes, workplace violence experienced by healthcare workers in healthcare settings is one of the problems that remains common to all countries. Due to its subjective nature and different perceptions, the lack of a single and clear definition of workplace violence, the wide variety of behaviours that can be included under the heading of workplace violence, and the fact that incidents of workplace violence in healthcare are often underreported, it is quite difficult to determine the true figures of workplace violence. The aim of this study, together with future studies, can help to reduce the difficulties mentioned. The reason for choosing the scale to be developed in this study is the belief that it will contribute to overcoming these difficulties. This is because the questions of the scale—defining violence, assessing the frequency of violence, assessing the impact of violence on the individual and society, examining the risk factors that reveal violence, examining the reasons for not reporting violence to the legal authorities and questioning the effectiveness of measures taken to reduce violence—are addressed under the subheadings as listed.

In the light of the above literature, the aim of this study is to develop a scale to overcome the problems experienced by healthcare decision makers in assessing violence in the workplace and to ensure that they implement appropriate interventions. In this context, we have formulated the following research question: Is the Workplace Violence Scale in Healthcare a valid and reliable measurement tool for Turkish healthcare workers? Consequently, this study aimed to cross-culturally adapt and psychometrically validate the Workplace Violence Scale in Healthcare for the Turkish population.

## 2. Materials and Methods

### 2.1. Methods

The study was initiated by obtaining permission from the authors who developed the survey questions for the Turkish adaptation of the Development and Validation of a Questionnaire to Evaluate Workplace Violence in Healthcare Settings survey developed by Kumari et al. (2021) [44]. Ethical committee approval for the study was then obtained from the Sakarya University Ethics Committee Presidency in its meeting on 15 July 2023, number 59/07 with the number E-61923333-050.99-261191. After the approval of the ethics committee, the approval of the study was obtained from the Provincial Health Directorate Scientific Strategy Development Center, where the health facilities where the study was conducted were affiliated, with the number E-65530689-799-247281086 and dated 14 June 2024.

The authors of the original scale were contacted by e-mail, and their permission was obtained to translate and adapt the scale into Turkish according to the principle of Brislin’s forward and backward questionnaire translation procedure. Initially, four bilingual authors familiar with the content carried out the forward translation (from English to Turkish). The translations obtained were evaluated, and common expressions and translations that differed were re-examined. The Turkish translation of the scale on which agreement was reached as a result of the evaluation was subjected to a backward translation (from Turkish to English) by three other bilingual experts. A panel of five experts fluent in both languages, including psychologists, linguists, psychiatrists and psychometrists, then reviewed the Turkish to English translation and the English to Turkish translation. They carefully compared each item with its counterpart in the original English version. The experts were also asked to review the content of the scale and check its cultural appropriateness for the Turkish population. During the language equivalence translation phase, the translators translated the scale as a group, and the researchers provided semantic guidance. The translators were provided with materials in the target language. The panel assessed the content relevance, conceptual consistency and practical applicability of the scale, ensuring that it was compatible with Turkish cultural norms while maintaining the integrity of the original constructs. The final Turkish version of the scale was pretested with 30 healthcare professionals, who provided positive feedback on its clarity and wording. No revisions were required, and the final version was adopted for the study.

The study was administered to 30 participants in their native language, and 2 weeks later, the translated Turkish version of the study was administered. The language validity coefficient of the scale was obtained by looking at the relationship between the scores obtained from the two scales [45]. The results of the correlation analysis of the scale scores are presented in Table 1.

As can be seen in Table 1, it was found that there was a high level of correlation between the translated scale and the scores obtained from the original language scale for each factor. Since the correlation data were at a high level, it was accepted that the language structure of the scale was appropriate.

To assess the content validity of the scale, the opinions of experts in the field were sought. Ten experts in the field were asked to rate the statements of the scale. At this stage, the experts were asked to give their opinion on the statements as “appropriate-item should be slightly revised-item should be seriously revised-item is not appropriate”. According to the opinions received from the experts, the Davis technique was used to calculate the Content Validity Index (CVI) value. In this rating criterion, the scores were as follows: “A = not appropriate, B = item needs to be changed appropriately, C = appropriate but minor changes are needed, D = appropriate”. The CVI value was expected to be greater than 0.80 [45]. The formula “CVI = number of experts who selected options (C) and (D)/total number of experts” was used, and the content validity of the study was ensured.

After language and scope validity studies and legal approvals were obtained, the study was started, and 191 participants for the pilot study of the scale and 632 participants for the final sample study were randomly selected on a voluntary basis from a total of 3726 healthcare workers employed in a fully equipped district and city hospital providing general diagnostic and treatment services. The following criteria were established as inclusion criteria for study participants: working in a public hospital, providing healthcare services, participating in the study voluntarily, not being diagnosed with a psychiatric disorder, not having committed a violent act (no criminal record), having face-to-face communication with the patient or the patient’s relative while providing healthcare services, and not working as an administrative/managerial person. The research model was the relational screening method. The relational screening model is a screening approach that aims to determine the existence of a common change between two or more variables. The relational screening model tries to determine whether the variables change together, and, if there is a change, how it happens [46]. The sample size was determined by considering the number of participants required for different universe sizes (N = 384) [47]. In this study, 632 participants were reached, and since the participants with extreme data sets were removed from the sample, the data analysis was carried out using the data of 627 participants. The data form used in the study was administered to the participants online using the Google survey method. The data form took an average of 20 min to complete. Data were collected between July and October 2024.

A pilot study was conducted to test the construct validity of the scale. The pilot study was conducted online with the participation of 191 healthcare workers. The data obtained from Google Forms were transferred to SPSS v.25 using Microsoft Excel 2021, and then the construct validity of the scale was tested. In the construct validity testing phase, an Exploratory Factor Analysis (EFA) of the scale was conducted using the data obtained from the pilot study. As a result of the evaluation, it was concluded that the Workplace Violence Scale in Healthcare Institutions has five subdimensions, and these subdimensions were named as frequency of workplace violence, impact of workplace violence on the individual, reasons for not reporting workplace violence, strategies to prevent workplace violence and risk factors that increase workplace violence. The final study was conducted in the study in which the pilot study was completed. The scale was also subjected to confirmatory factor analysis (CFA) to verify construct validity. The AMOS programme 25.0 was used for the confirmatory factor analysis. In order to show the acceptability in the analysis results, the fit indices were examined. When the fit indices obtained as a result of CFA were analysed, it was found that the χ^2^/sd value was 3.325, which was at an “acceptable fit” level, and the RMSEA value was 0.061, which was at an “acceptable fit” level [48].

The study used internal consistency coefficients and time-invariance methods as reliability methods. The reliability of the confirmatory factor analysis model was assessed by looking at the “Average Explained Variance (AVE)” and “Composite Reliability (CR)” values. As the CR value was above the reference value of 0.70 and the AVE value was above the reference value of 0.50, the reliability and convergent validity of the CFA model were ensured [47]. To ensure time invariance, participants were asked to code the first two letters of their first and last name or write their email address on the data forms in order to reach the same health worker, and the test was repeated with 100 health workers who participated in our main study at three-week intervals. The test results were evaluated using the Pearson correlation test.

### 2.2. Data Analysis

SPSS 25.0 and AMOS were used to analyse the data. In the study, frequency and percentage analyses of socio-demographic characteristics were performed with a 95% confidence level, “Cronbach’s alpha” analysis was performed to assess the reliability of the scale, and “Pearson correlation” analyses were performed to assess the existence of a relationship between measurements and the strength and direction of this relationship.

### 2.3. Data Collection Tool

#### 2.3.1. Sociodemographic Data Form

Questions such as the participant’s age, gender, level of education, marital status, occupation, length of work experience, duty status, whether or not they were exposed to violence during their work experience, the unit where they were exposed to violence, and the time period in which the violent incident occurred were created by the researchers as a result of the literature review.

#### 2.3.2. Workplace Violence Scale in Healthcare

Development and Validation of a Questionnaire to Evaluate Workplace Violence in Healthcare Settings is a questionnaire developed by Kumari et al. (2021) using data from 213 healthcare workers (including doctors and other healthcare workers from different departments such as emergency medicine, medicine, psychiatry, nursing, obstetrics and gynaecology). The questionnaire consists of five factors and 37 items. The questionnaire was named A: Forms of violence, B: Impact of incidents of violence, C: Reporting of incidents, D: Mitigation strategies, and E: Risk factors associated with incidents of workplace violence [44].

## 3. Results

### Results of the Pilot Study

The pilot study of the research was conducted with a total of 191 participants, and the results for the socio-demographic characteristics of the participants are shown in Table 2.

In Table 3, items B1, C1 and D1 were removed from the scale because their correlation values with other items were below 0.30 and they reduced the reliability level. As can be seen in Table 3, since the correlation values of the remaining items in the scale were not below 0.30, it was decided that there was no need to remove more items, and the reliability level of the scale was determined to be high (C.Alpha = 0.946).

The prerequisite for carrying out factor analysis is that the sample size is sufficiently large. To this end, the Kaiser–Meyer–Olkin (KMO) test was carried out first, followed by the Barlett sphericity test, in order to verify the existence of a relationship between the variables. As can be seen in Table 3, the KMO value was greater than 0.60, and the Barlett sphericity test was significant (*p* < 0.05).

In order to decide whether an item should remain in the scale, the factor loading value should be higher than 0.45. At the same time, the overlap of the items was examined, and their loading on a factor was also taken into account. As a result of the factor analysis, it was found that there was a 5-factor structure, and the factor loadings were between 0.537–0.892. It was seen that the total variance value of the scale explained by these factors was 73.405%, and as this value was above 0.40, it showed that it had sufficient explanatory power. As the scale had more than one factor, a “varimax” vertical rotation was performed. After the rotation, it was found that Factor 1 alone explained 24.584% of the scale with an eigenvalue of 8.359, and when the items under the relevant factor were examined, the factor was named “Risk Factors Increasing Workplace Violence” and the reliability of the factor was found to be high at 0.956. It was determined that Factor 2 alone explained 20.255% of the scale with an eigenvalue of 6.887, and when the items under the relevant factor were examined, the factor was named “Workplace Violence Prevention Strategies”, and the reliability level of the factor was determined to be high at 0.940. It was determined that Factor 3 alone explained 12.809% of the scale with an eigenvalue of 4.355, and when the items under the relevant factor were examined, the factor was named “Reasons for not reporting workplace violence”, and the reliability level of the factor was determined to be high at 0.907. It was found that Factor 4 alone explained 11.352% of the scale with an eigenvalue of 3.86, and when the items under the relevant factor were examined, the factor was named “Impact of Workplace Violence on the Individual”, and the reliability level of the factor was found to be high at 0.913. It was found that Factor 5 alone explained 4.405% of the scale with an eigenvalue of 1.498, and when the items under the relevant factor were examined, the factor was named “Frequency of Violence at Work”, and the reliability of the factor was found to be at an acceptable level of 0.642.

**Figure 1:**
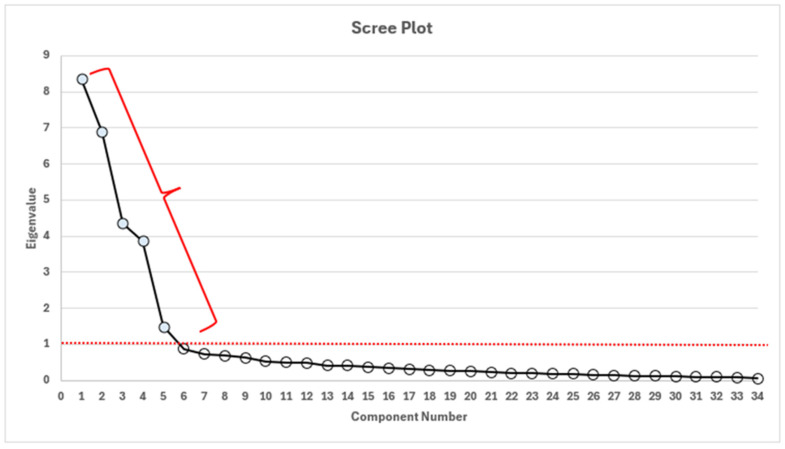
Slope plot of the Workplace Violence Scale in Healthcare.

In order to make the most accurate decision about the factor structure of the scale, a ‘scree plot’ of the observations was examined. When the scree plot of the Workplace Violence Scale in Healthcare was evaluated, it was seen that the break occurred after the 5th factor, when the observation value of the scale fell below 1. The scree plot of the Workplace Violence Scale in Healthcare was evaluated. The scree plot is a graph where the amount of information (eigenvalue) in the scale is calculated for each new component, with the eigenvalue on the vertical axis and the number of components on the horizontal axis. The relationship between the number of factors (Component Number) and the eigenvalue in the graph can give an idea of the ideal number of factors. The eigenvalue is the amount of information contained in each factor. The number of factors that contain most of the information and at the same time do not contain a significant amount of information can be interpreted as the ideal number of factors. When the graph is examined, there is a structure that contains a high amount of information up to the 5th factor, therefore there is a rapid decrease in the eigenvalue. The bend between the 5th and 6th factors can be interpreted as the factors to be created after this point will contain a very small amount of information. Therefore, based on the slope scatter plot, it can be said that the ideal number of factors should be 5, and the factors created after the 5th factor will not contain significant information. The results are presented in Figure 1.

To identify the lower and upper 27% of participants, the total score on the Workplace Violence Scale in Healthcare was ranked from lowest to highest. The lowest 52 and highest 52 values of the ranked scores were examined (Table 4). It was found that the lower and upper 27% values used to discriminate the items were significant for all items (*p* < 0.05).

Since the item–total relationship value was above 0.30 for all items, it was determined that the measurement power of the items was sufficient. When Table 5 was examined, it was found that the relationships between the scale items and the average score obtained from the scale varied between 0.406–0.788 and that the relationships were statistically significant (*p* < 0.05). According to this result, it was concluded that there was no problem in the consistency of the items with each other.

As a result of the final study of the research, a total of 627 participants were reached, and it was determined that 64.90% of the participants were female (*n* = 407) and 35.10% were male (*n* = 220), and they were between the ages of 36–45 (41.10%; *n* = 258), married (72.60%; *n* = 455) and undergraduates (42.30%; *n* = 265). The results are shown in Table 6.

Of the participants, 64.90% were female and 35.10% were male, 41.14% of the participants were between 36 and 45 years old, 72.60% were married, 42.30% had a Bachelor’s degree, and 40.20% were nurses, midwives, or health officers. Other characteristics are shown in Table 6. Table 7 analyses the general occupational characteristics of the participants and their experiences of violence in the workplace. According to this, the participants had mostly worked for 16 years or more (%43.20; *n* = 271), they were on duty (%57.30; *n* = 359), the participants who were on duty mostly worked between 4–7 shifts (%39.80; *n* = 143), they were exposed to physical, verbal, psychological or sexual violence by patients and their relatives during their work (%60.80; *n* = 381), they were exposed to verbal violence (%77.40; *n* = 295), they were exposed to violence in the outpatient department (%46.50; *n* = 177), they had worked in the area where they were exposed to violence for 1–5 years (%45.70; *n* = 174), the violence occurred during working hours (08.00–17.00) (%64.00; *n* = 244), the perpetrator was a relative of the patient (%64.00; *n* = 244), the person who perpetrated the violence was male (%67.20; *n* = 256), they did not report violent incidents to the police (%68.50; *n* = 261), they were a little worried if they were exposed to violence (%54.10; *n* = 339), and they thought that violent incidents were partly preventable (%53.40; *n* = 335).

**Figure 2:**
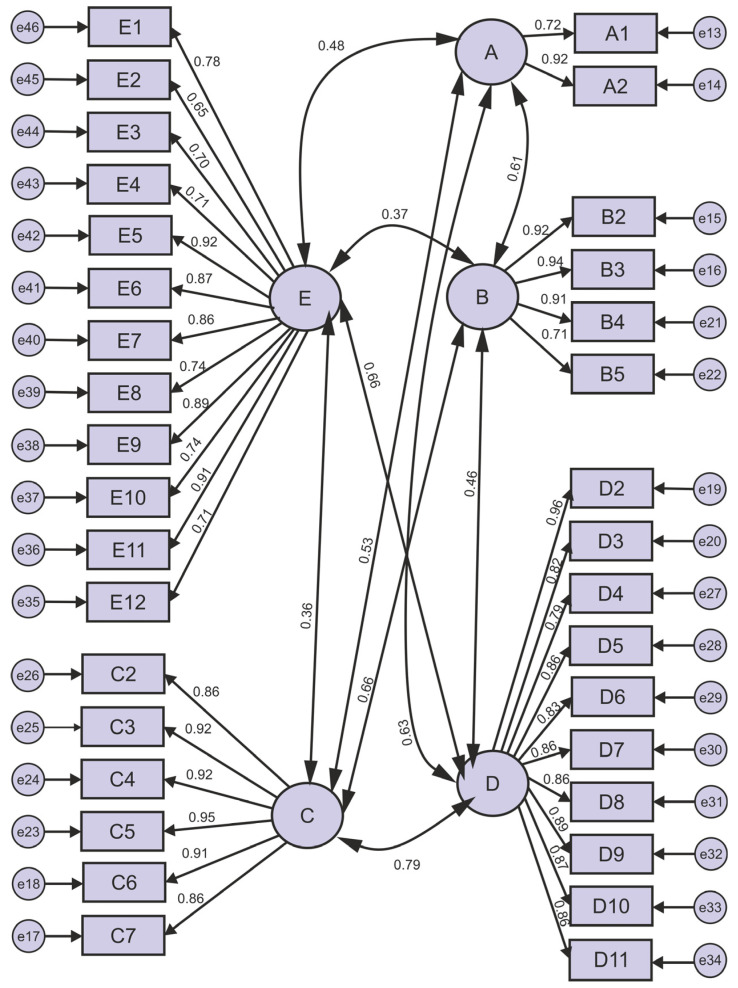
A: Frequency of workplace violence, B: Impact of workplace violence on individuals, C: Reasons why workplace violence goes unreported, D: Strategies to prevent workplace violence, E: Risk factors that increase workplace violence.

The standardised beta coefficients of the CFA analysis carried out to verify the explained factor structure are presented as a path diagram in Figure 2. Looking at Figure 2, according to the CFA results of the scale of workplace violence in healthcare institutions, no modification was necessary, as the criteria of conformity were at the desired level in the first stage.

The t-values and significance results of the factor loadings of the scale are presented in Table 8. As the factor loadings of the items of the scale were found to be between 0.65 and 0.97 as a result of the Confirmatory Factor Analysis (CFA) (Figure 2), these values are acceptable. The t-values, which express the level of statistical significance of the relationships between the items and the latent variables, were found to be significant at the *p* < 0.05 level, and all values were greater than 2.58 (Table 8).

The main study result of the scale on which the fit study was conducted indicated that the fit criteria were in the acceptable and good fit ranges. When the values of the fit criteria obtained as a result of the CFA were examined, it was found that the ratio of the most important fit value, the χ^2^ value, to the sd value was 3.325 at an acceptable level of fit, and the RMSEA value was 0.061 at an acceptable level of fit. As the other fit criteria were also found to be within the acceptable and good fit ranges, the results show that the explained factor structure was confirmed. The results are presented in Table 9.

The reliability of the CFA model was tested by looking at the Average Variance Explained (AVE) and Composite Reliability (CR) values. As the CR values in Table 10 were found to be above the threshold value of 0.70, and the AVE values were found to be above the threshold value of 0.50, it was determined that the reliability and convergent validity of the CFA model were ensured. In addition, it was determined that the reliability levels of the general dimensions and subdimensions of the scale were sufficient.

It can be seen that there was a positive and significant relationship between the test-retest results evaluated by Pearson correlation analysis. Since the correlation levels were high, it was concluded that there is no change in the scale over time. The results are shown in Table 11.

## 4. Discussion

In this study, the Turkish adaptation of the Workplace Violence Scale in Healthcare, validity and reliability analyses were conducted. As a result of the analyses, the scale, a five-dimensional, 37-item Likert-type self-report instrument, was found to be a valid and reliable measurement tool with psychometric properties. The scale was found to have a high internal consistency coefficient (0.94) and stability over time (0.97).

For the validity and reliability analyses of the Workplace Violence Scale in Healthcare, firstly, item analysis, item scale total correlation, item discrimination analysis, Explanatory Factor Analysis (EFA) and reliability analyses were carried out with the data obtained as a result of the pilot study. The developed data form was administered to 191 people, and a pilot study was conducted. There are several important purposes for conducting a pilot study. Firstly, in order to ensure the structural validity of the scale, EFA was used to eliminate invalid items and ensure that the scale had structural validity characteristics with five subdimensions. Next, the reliability levels of the scale and subdimensions were tested. Subdimensions should be at a level that allows for reliable measurement. Therefore, Cronbach’s alpha reliability analysis was applied during the pilot application. Confirmatory Factor Analysis (CFA) is a statistical technique used by the researcher to understand which variables in the data set form consistent subsets independently of others. Variables that are related to each other, but not largely related to other subsets of variables, are grouped together as factors. Factors are thought to reflect the underlying processes that generate correlations between variables [49]. During the exploratory factor analysis, the Kaiser–Meyer–Olkin (KMO) sampling adequacy measure and Bartlett sphericity tests were examined. The sampling adequacy of the items was determined using the KMO sampling adequacy measure, and the Bartlett sphericity test was used to determine whether the items were sufficiently related to measure a phenomenon. The KMO sampling adequacy measure is applied to determine the adequacy of a scale consisting of k items in measuring a phenomenon, and the value is expected to be greater than 0.5 [50]. When this value approaches 1, it is assumed that the adequacy of the scale increases. In the pilot study conducted with 119 people, the KMO statistic was found to be 0.924, which means that this scale met the sampling adequacy condition at a high level (KMO > 0.80). The Bartlett sphericity test is a method that examines the relationship between the items of a scale [50]. If the results of the Bartlett sphericity test are found to be significant, factor analysis can be applied to these data. It has been proven that the significance value of the Bartlett sphericity test statistic has a sufficient level of relationship with the scale items.

As a result of the appropriate data obtained from the Bartlett and KMO test statistics, the stage of determining the factors of the scale was started. For this purpose, the literature was used in the stage of determining the scale items, the eigenvalues in the scree plot and the total variance explained by the factors. The eigenvalues given by the scree plot, which are between 1 and 2, indicate the appropriate number of factors [51]. The total variance explained by the factors explains the extent to which the subdimensions explain the change in the scale. The fact that the total variance explained is more than 50% indicates that the scale has sufficient variance explanation [50]. The correlation of each item in the scale with the items other than itself should be above 0.30 [52]. Therefore, the correlation values of questions numbered B1, C1, and D1 with other items in the scale were found to be below 0.30 and to reduce the reliability level, so they were removed from the scale. As the correlation value of the remaining items in the scale with other items was not below 0.30, it was decided that there was no need to remove any more items from the scale. After removing the items from the scale, it was found that the reliability of the scale was high (Cronbach alpha = 0.946). Cronbach alpha reliability analysis 1 > X > 0.90 is accepted as “perfect reliability” [50]. The Slope Sprinkle Plot was used in the factor analysis, and the results are shown in Figure 1. The Slope Sprinkle Plot is a graph where the amount of information (eigenvalue) in the scale is calculated for each new component, with the eigenvalue on the vertical axis and the number of components on the horizontal axis. The eigenvalue of each new component in the scale decreases. Therefore, the appropriate number of factors will be in the areas where the new components have relatively small eigenvalues. The range where the eigenvalue is below 2 and above 1 indicates the appropriate factor number. Since the information contained by the new factors is quite small in the factor numbers above this range, it can be said that they do not make a significant contribution to the scale. It is known that when the eigenvalue is above 2, there is a loss of information in the scale due to the new factors not being considered [51]. The relationship between the number of factors (component number) and the eigenvalue in the graph can give an idea of the ideal number of factors. The eigenvalue is the amount of information carried by each factor. The number of factors that carry the largest amount of information, and at the same time do not carry a significantly low amount of information, can be interpreted as an ideal. Looking at the graph, there is a structure that contains a high amount of information up to the 5th factor; therefore, there is a rapid decrease in the eigenvalue. The bend between the 5th and 6th factors can be interpreted, as the factors to be created after this point contain a very small amount of information. Therefore, based on the slope scatter plot, it can be said that the ideal number of factors should be 5, and the factors created after the 5th factor will not contain significant information.

To control for item discrimination, groups below and above 27% are identified, and item discrimination scores are determined based on the responses of these [53]. In the current study, the responses of the lowest 50 and the highest 50 individuals were examined, and it was found that all items were significant.

As a result of CFA and reliability analysis during the pilot application, the scale was found to have structural validity and reliability characteristics with five subdimensions and 34 items. After this stage, the structural validity and reliability level of the scale will be verified with a second sample. In order to verify the structural validity and reliability of the scale in a second sample, data were collected from 632 samples. Five of the observations were excluded from the study because they contained extreme values, and the analyses were continued with 627 observations to support the results of the pilot study. CFA was used to verify the factor structure that emerged from the explanatory factor analysis conducted in the pilot study with the second sample.

CFA is an examination/control and testing method used to control factor structures revealed by using certain sources, factor structures previously accepted in original scales, or factor structures revealed in preliminary studies [50]. According to the fit criteria of the applied confirmatory factor analysis, sufficient reliability results were obtained. As a result of the CFA conducted with the data of the main study, it was found that the factor loadings of the items belonging to the scale were between 0.65–0.97. The t-values, which express the level of statistical significance of the relationships between the items and the latent variables, were found to be significant at the *p* < 0.05 level, and it was seen that all the values were greater than 2.58. There are many methods used to assess the fit of the data during confirmatory factor analysis. The first of these methods is the χ^2^/sd ratio, where a ratio of less than 5 indicates an acceptable level of fit and a ratio of less than 3 indicates a perfect level of fit [54]. As the χ^2^/sd value of the current study was found to be 3.325, it could be seen that the scale had an acceptable level of fit. Another criterion, the RMSEA value, is expected to be less than 0.05 or between 0.05 and 0.08. Considering that the RMSEA value of the current study was 0.061, it can be said that it had an acceptable level of fit. CFI is the model that accepts that there is no relationship between the variables, and this value between 0.95 and 1 indicates a perfect fit [55]. Since the CFI value in the current study was 0.946, it can be seen that the model had a perfect fit. When the NFI and IFI values, which are used to eliminate the possibility of the effect of sample size, are between 0.95 and 1, a perfect fit can be said to exist. The NFI value in the current study was found to be 0.925, and the IFI value was found to be 0.947, and therefore, it can be said that it had a perfect fit. It is said that the degree of fit of the model increases as the RMR value approaches 0 and that it has a perfect fit value between 0 and 0.05 [56]. The RMR value in the current study was found to be 0.024, proving that the model had a good fit.

The AVE value, which reveals the relationship between the items and the factors they belong to, is expected to be greater than 0.50 [57]. In the current study, the lowest AVE value was considered to be 0.63. This shows that the convergent validity of the scale was achieved. The composite reliability coefficient (CR) is also expected to be above 0.70 [58]. In the current study, the CR values of the factors varied between 0.80 and 0.95. When Cronbach’s alpha values and CR values were evaluated, it could be seen that the scale was a reliable scale with all its subdimensions.

In order to show the consistency of the variables to be measured over time, the participants were contacted again after 3 weeks, and the scale was completed again, and a test-retest analysis was carried out. Test-retest reliability is a method of security analysis obtained by comparing the same participant’s scores on the same scale at different times [59]. In order to evaluate the findings, the Pearson correlation analysis, which was used to reveal the relationship between the two variables, showed that the r values were 0.80 for the ‘frequency of workplace violence’ factor, 0.88 for the ‘impact of workplace violence on the individual’ factor, 0.94 for the ‘reasons for not reporting workplace violence’ factor, 0.938 for the ‘strategies to prevent workplace violence’ factor and 0.93 for the ‘risk factors that increase workplace violence’ factor. A Pearson correlation coefficient (r) greater than 0.80 indicates a very high correlation. Looking at the current results of the scale, the values obtained showed that it was a consistent scale over time.

## 5. Conclusions

As a result of the research conducted with 627 participants to develop a valid and reliable scale that would reveal workplace violence against healthcare workers and provide measurement together with its subdimensions, a valid and reliable measurement tool that revealed workplace violence was obtained. The Cronbach Alpha coefficient was highly reliable at 0.94. The CR values of the scale consisting of five subdimensions and 37 items were above 0.70, and the AVE values were above 0.50. At the same time, with the test-retest method conducted to reveal the stability of the scale against time, it was seen that the subdimension coefficients were between 0.80 and 0.94, and 0.97 for the entire scale. This shows that it has an excellent level of reliability in its durability against time. In addition, the Workplace Violence Scale in Healthcare provides a comprehensive assessment with the subdimensions of “Frequency of Workplace Violence”, “Impact of Workplace Violence on the Individual”, “Reasons for Not Reporting Workplace Violence (to Legal Authorities)”, “Strategies to Prevent Workplace Violence”, and “Risk Factors Increasing Workplace Violence”. Section A of the scale (frequency of workplace violence) asks whether healthcare workers have experienced verbal and physical violence while providing healthcare services and within the healthcare institution, and if so, how often. Section B of the scale (personal impact of workplace violence) asks whether the personal health (physical and mental), family life and social life of health workers have been affected by the violence they have experienced and, if so, to what extent (five-point Likert scale from not affected at all to very affected). In the C section of the scale (reasons for not reporting workplace violence (to legal authorities)), healthcare workers are asked to rate their level of comfort or confidence in reporting violent incidents they have experienced to higher authorities, and to rate the importance of factors that are thought to be important in not reporting violent incidents to authorities and that have been prepared according to the information in the literature. The D section of the scale (strategies to prevent violence in the workplace) asks healthcare workers to rate the extent to which strategies that can be useful in preventing violent incidents in the workplace are/could be useful in controlling violence in the workplace. The E section of the scale (risk factors that increase violence in the workplace) assesses various risk factors associated with violence in healthcare settings and the level of importance of these risk factors. As a result of all the analyses, it was concluded that the Workplace Violence Scale in Healthcare is a valid and reliable psychometric assessment tool.

## Figures and Tables

**Table 1:** Language validity measurement results.

Measurements		Language Validity
Forms of Violence	r	0.731
	*p*	0.001 **
Impact of Violent Incidents	r	0.925
	*p*	0.001 **
Reporting Incidents	r	0.931
	*p*	0.001 **
Mitigation Strategies	r	0.947
	*p*	0.001 **
Risk Factors Related to Workplace Violence	r	0.804
	*p*	0.001 **

** *p* < 0.01.

**Table 2:** Distribution of socio-demographic characteristics of pilot study participants.

Demographic Characteristics	Group	*n*	%
Gender	Female	141	73.82
	Male	50	26.18
Age	18–25 years	4	2.09
	26–35 years	42	21.99
	36–45 years	88	46.07
	46+ years	57	29.85
Marital Status	Married	150	78.53
	Single	41	21.47
Educational Status	Middle School	10	5.24
	High School	27	14.14
	Associate Degree	19	9.95
	Bachelor’s Degree	98	51.31
	Master’s Degree	26	13.61
	Doctorate	11	5.75
Job	Doctor	15	7.85
	Nurse/Midwife/Health officer	96	50.26
	Cleaning staff	24	12.57
	Medical secretary	24	12.57
	Laboratory/Radiology technician	13	6.81
	Other (dietician, psychologist, audiologist, etc.)	19	9.94
	Total	191	100.00

It was determined that 73.82% of the individuals included in the study were female (*n*: 141), 26.18% were male (*n*: 50), the individuals were mostly between the ages of 36–45 (*n*: 88; 46.07%), married (*n*: 150, 78.53%), had a bachelor’s degree (*n*: 98, 51.31%) and were nurses/midwifes/health officers (*n*: 96, 50.26%).

**Table 3:** Exploratory factor analysis, reliability levels and item analysis results of the workplace violence scale in healthcare.

Item No	Factors					Item-Scale Correlation
	1	2	3	4	5	
E2	0.681					0.566
E8	0.732					0.600
E1	0.729					0.761
E12	0.753					0.633
E3	0.799					0.606
E7	0.804					0.684
E10	0.774					0.662
E4	0.820					0.646
E6	0.856					0.643
E9	0.853					0.651
E5	0.857					0.704
E11	0.892					0.691
D4		0.594				0.444
D2		0.645				0.723
D3		0.748				0.493
D7		0.812				0.618
D6		0.801				0.568
D10		0.831				0.596
D9		0.865				0.629
D8		0.848				0.650
D5		0.845				0.539
D11		0.878				0.555
C7			0.748			0.349
C2			0.732			0.640
C6			0.784			0.494
C3			0.828			0.480
C4			0.869			0.501
C5			0.875			0.546
B5				0.814		0.484
B2				0.761		0.693
B3				0.861		0.562
B4				0.890		0.537
A2					0.537	0.389
A1					0.667	0.423
Reliability	0.956	0.94	0.907	0.913	0.642	0.946
Eigenvalue	8.359	6.887	4.355	3.86	1.498	
Explained Variance (%)	24.584	20.255	12.809	11.352	4.405	73.405

KMO: 0.913; Bartlett’s Test of Sphericity = χ^2^ (561) = 6117.777; *p* = 0.001 < 0.05.

**Table 4:** Testing the discriminatory power of items on the Workplace Violence Scale in Healthcare by 27% lower and upper groups.

Item	t	*p*	Item	t	*p*
A1	−7.131	0.001 *	D7	−9.245	0.001 *
A2	−5.607	0.001 *	D8	−10.058	0.001 *
B2	−15.447	0.001 *	D9	−7.554	0.001 *
B3	−9.507	0.001 *	D10	−8.650	0.001 *
B4	−10.311	0.001 *	D11	−7.677	0.001 *
B5	−9.355	0.001 *	E1	−18.338	0.001 *
C2	−12.286	0.001 *	E2	−10.358	0.001 *
C3	−8.044	0.001 *	E3	−10.565	0.001 *
C4	−10.587	0.001 *	E4	−13.377	0.001 *
C5	−11.053	0.001 *	E5	−13.115	0.001 *
C6	−8.691	0.001 *	E6	−11.222	0.001 *
C7	−5.900	0.001 *	E7	−12.429	0.001 *
D2	−15.761	0.001 *	E8	−12.303	0.001 *
D3	−8.358	0.001 *	E9	−10.547	0.001 *
D4	−7.806	0.001 *	E10	−11.980	0.001 *
D5	−7.724	0.001 *	E11	−10.761	0.001 *
D6	−9.058	0.001 *	E12	−13.894	0.001 *

* *p* < 0.05; t: Independent samples *t*-test was performed.

**Table 5:** Scale items of the Workplace Violence Scale in Healthcare and scale total correlation values.

Item	r	*p*	Item	r	*p*
A1	0.473	0.001 *	D7	0.642	0.001 *
A2	0.431	0.001 *	D8	0.675	0.001 *
B2	0.723	0.001 *	D9	0.651	0.001 *
B3	0.602	0.001 *	D10	0.622	0.001 *
B4	0.579	0.001 *	D11	0.581	0.001 *
B5	0.531	0.001 *	E1	0.788	0.001 *
C2	0.676	0.001 *	E2	0.597	0.001 *
C3	0.516	0.001 *	E3	0.632	0.001 *
C4	0.537	0.001 *	E4	0.670	0.001 *
C5	0.578	0.001 *	E5	0.725	0.001 *
C6	0.533	0.001 *	E6	0.667	0.001 *
C7	0.406	0.001 *	E7	0.705	0.001 *
D2	0.752	0.001 *	E8	0.628	0.001 *
D3	0.529	0.001 *	E9	0.673	0.001 *
D4	0.486	0.001 *	E10	0.685	0.001 *
D5	0.568	0.001 *	E11	0.710	0.001 *
D6	0.597	0.001 *	E12	0.658	0.001 *

* *p* < 0.05; r: Pearson correlation analysis.

**Table 6:** Distribution of socio-demographic characteristics of final survey respondents.

Demographic Characteristics	Group	*n*	%
Gender	Female	407	64.90
	Male	220	35.10
Age	18–25 years	48	7.65
	26–35 years	179	28.54
	36–45 years	258	41.14
	46+ years	142	22.67
Marital Status	Married	455	72.60
	Single	172	27.40
Educational Status	Middle School	22	3.50
	High School	84	13.40
	Associate Degree	87	13.90
	Bachelor’s Degree	265	42.30
	Master’s Degree	89	14.20
	Doctorate	80	12.70
Job	Doctor	102	16.30
	Nurse/Midwife/Health officer	252	40.20
	Medical secretary	102	16.30
	Cleaning staff	59	9.40
	Laboratory/Radiology technician	53	8.50
	Other (dietician, psychologist, audiologist etc.)	59	9.30
	Total	627	100.00

**Table 7:** General occupational characteristics of healthcare workers and experiences of violence at work.

Professional Qualifications	Groups	*n*	%
Professional duration	Less than 1 year	46	7.30
	1–5 years	99	15.80
	6–10 years	127	20.30
	11–15 years	84	13.40
	16+ years	271	43.20
Working on shifts	Yes	359	57.30
	No	268	42.70
# Number of monthly shifts	1–3	82	22.90
	4–7	143	39.80
	8+	134	37.30
Exposure to violence at work from patients and their relatives	Yes	381	60.80
	NO	246	39.20
# Type of violence exposed	Sexual and physical V.	86	22.60
	verbal V.	295	77.40
#Health unit where the violent incident occurred	Polyclinic	177	46.50
	Inpatient service	84	22.00
	Emergency service	88	23.10
	Other	32	8.40
# Working hours in the health unit where violence was experienced	Less than 1 year	43	11.30
	1–5 years	174	45.70
	6–10 years	80	21.00
	11–15 years	44	11.50
	16+ years	40	10.50
# Time of the violent incident	Working hours (08–17)	244	64.00
	Weekdays (17–08)	83	21.80
	Weekends (08–08)	54	14.20
# The person who commits the act of violence	Patient	137	36.00
	Patient relative	244	64.00
# Gender of the person committing the act of violence	Female	65	17.10
	Male	256	67.20
	Men and women together	60	15.70
Status of making legal notification regarding violent incident	Yes	120	31.50
	No	261	68.50
Concern about being exposed to violence while working in a hospital	I have no concerns	71	11.30
	I am somewhat concerned	339	54.10
	I am very concerned	217	34.60
Belief that violent incidents are preventable	Absolutely yes	242	38.60
	Partially	335	53.40
	Absolutely unavoidable	50	8.00
# The variation can be seen in the number *n*.	Total	627	100.00

**Table 8:** T-values of the factor loadings of the CFA model.

Item No	Factor	Factor Loading	t Value	*p*
A1	A	0.72	-	0.001 *
A2	A	0.92	18.092	0.001 *
B2	B	0.92	-	0.001 *
B3	B	0.94	41.713	0.001 *
B4	B	0.91	38.517	0.001 *
B5	B	0.71	22.508	0.001 *
C2	C	0.86	29.408	0.001 *
C3	C	0.92	33.395	0.001 *
C4	C	0.92	33.176	0.001 *
C5	C	0.95	35.829	0.001 *
C6	C	0.91	32.414	0.001 *
C7	C	0.86	-	0.001 *
D2	D	0.97	-	0.001 *
D3	D	0.82	32.682	0.001 *
D4	D	0.79	30.304	0.001 *
D5	D	0.86	38.145	0.001 *
D6	D	0.83	34.149	0.001 *
D7	D	0.83	34.414	0.001 *
D8	D	0.86	38.33	0.001 *
D9	D	0.89	41.61	0.001 *
D10	D	0.87	39.309	0.001 *
D11	D	0.86	37.989	0.001 *
E1	E	0.78	19.107	0.001 *
E2	E	0.65	16.065	0.001 *
E3	E	0.70	17.177	0.001 *
E4	E	0.71	17.471	0.001 *
E5	E	0.92	22.647	0.001 *
E6	E	0.87	21.248	0.001 *
E7	E	0.86	20.999	0.001 *
E8	E	0.74	18.198	0.001 *
E9	E	0.89	21.923	0.001 *
E10	E	0.75	18.293	0.001 *
E11	E	0.91	22.292	0.001 *
E12	E	0.71	-	0.001 *

* *p* < 0.05.

**Table 9:** Results of the criteria for compliance with the Workplace Violence Scale in Healthcare.

Compliance Criteria	Good Fit	Acceptable Fit	Research Result	Fit Level
χ^2^	1719.034			
Sd	517			
χ^2^/sd	≤3	≤5	3.325	Acceptable
RMSEA	0 < RMSEA < 0.05	0.05 ≤ RMSEA ≤ 0.10	0.061	Acceptable
RMR	0 ≤ RMR < 0.05	0.05 ≤ RMR ≤ 0.10	0.024	Good
NFI	0.95 ≤ NFI ≤ 1	0.90 ≤ NFI ≤ 0.95	0.925	Acceptable
CFI	0.95 ≤ CFI ≤ 1	0.90 ≤ CFI ≤ 0.95	0.946	Acceptable
IFI	0.95 ≤ IFI ≤ 1	0.90 ≤ IFI ≤ 0.95	0.947	Acceptable
RFI	0.95 ≤ RFI ≤ 1	0.90 ≤ RFI ≤ 0.95	0.919	Acceptable

**Table 10:** AVE and CR scores for subdimensions of the Workplace Violence Scale in Healthcare.

Factors	Reliability	CR	AVE
Forms of Violence	0.792	0.80	0.68
Impact of Violent Incidents	0.926	0.93	0.77
Reporting Incidents	0.963	0.96	0.81
Mitigation Strategies	0.964	0.97	0.74
Risk Factors for Workplace Violence	0.951	0.95	0.63
Scale Overall Cronbach’s Alpha: 0.968			

**Table 11:** Test-retest results for the stability over time of the subdimensions of the Workplace Violence Scale in Healthcare.

Measurements	Retest		
	*n*	r	*p*
Frequency of Workplace Violence	100	0.807	0.001 *
Impact of Workplace Violence on the Individual	100	0.882	0.001 *
Reasons for Workplace Violence Not Being Reported	100	0.945	0.001 *
Strategies to Prevent Workplace Violence	100	0.938	0.001 *
Risk Factors that Increase Workplace Violence	100	0.937	0.001 *
General	100	0.973	0.001 *

* *p* < 0.05; r: Pearson Correlation Analysis.

## Data Availability

The data presented in this study are available to all researchers.
